# Enhancing toxicological interpretation and reporting of *in vitro* high-throughput screening data

**DOI:** 10.3389/ftox.2026.1886801

**Published:** 2026-08-24

**Authors:** Bridgett N. Hill, Agnes L. Karmaus, Victoria Hull, Aswani Unnikrishnan, Nicole C. Kleinstreuer, Helena T. Hogberg

**Affiliations:** 1 Inotiv, Research Triangle Park, NC, United States; 2 General Dynamics Information Technology, Falls Church, VA, United States; 3 Applied Research Associates, Inc., Raleigh, NC, United States; 4 National Toxicology Program Interagency Center for the Evaluation of Alternative Toxicological Methods, National Institute of Environmental Health Sciences, Research Triangle Park, NC, United States

**Keywords:** annotation, ontology, Tox21, ToxCast, toxicological interpretation

## Abstract

**Introduction:**

A wide variety of in vitro high-throughput screening (HTS) assay data are publicly available and have the potential to serve as the foundation for the development of computational toxicology approaches. However, the sources of these data vary in the format and metadata they provide and the clarity of their relevance to toxicological endpoints. Thus, applying them to decisions about chemical effects remains a challenge. To overcome this challenge and help democratize HTS data, we curated and annotated HTS data specifically for toxicological interpretation within a regulatory endpoint context.

**Methods:**

Focusing on data from the publicly available ToxCast/Tox21 HTS program, a curation workflow was developed to identify the most reliable data by integrating metadata such as chemical quality control data, concentration–response curve-fitting robustness, assay-specific limitations, and potential technological interference. To this curated HTS (cHTS) dataset, we then applied structured vocabularies and expert review to develop molecular/cellular process annotations mapping HTS assays to toxicologically relevant regulatory endpoints. These data were also mapped to the Organization for Economic Co-operation and Development’s Harmonised Template 201 (OHT 201) reporting format to standardize reporting, rendering outputs amenable to regulatory submission.

**Results:**

The curated and annotated cHTS dataset supports data interoperability and allows these data to be searched, grouped, and visualized by regulatory endpoints (e.g., endocrine disruption, carcinogenicity, developmental toxicity, and systemic effects). The robust cHTS data and annotations can be retrieved from the publicly accessible National Institute of Health (NIH) Integrated Chemical Environment (NICE, https://ice.ntp.niehs.nih.gov/). Access through NICE allows these data to be optimally leveraged for a variety of uses, from identifying data gaps and providing mechanistic insights to confidently and reproducibly retrieving data, thus supporting their integration to build weight-of-evidence assessments for regulatory-relevant toxicological endpoints.

**Discussion:**

Developing an environment in which HTS data that are structured, accessible, and readily interpretable in a toxicological context, ensures that users can apply these evolving resources. Working toward consistent data summaries by leveraging established reporting formats helps bolster regulatory interpretation and adoption. Overall, the work summarized herein demonstrates how fit-for-purpose standardized curation, annotations, ontologies, ease of accessibility, and structured reporting can support data interoperability and the application of complex HTS data for regulatory application.

## 1 Introduction

Traditionally, *in vivo* animal testing has been employed to evaluate the toxicity of chemicals and drugs. However, these studies are time-consuming, require large numbers of animals, offer limited mechanistic insights, and often face challenges in translating results to humans. Consequently, many chemicals lack sufficient toxicity data. To increase and enhance the ethical and mechanistic insights into chemical-elicited toxicity, initiatives such as the 3Rs (Russell and Burch, 1959; Schechtman, 2002) and a report by the U.S. National Academy of Sciences (NRC, 2007) have called for the development and use of new approach methodologies (NAMs) in toxicology. Since these seminal publications and paradigms were initiated, an abundance of NAMs have been developed (Kavlock et al., 2018; Krewski et al., 2020; Richard et al., 2021). Most recently, frameworks for modernizing chemical hazard assessment and risk assessment have been the focus, discussing how to apply the diversity of information provided by these approaches (i.e., biological pathway modeling and mechanistic information to inform on modes of action) into safety assessments (van der Zalm et al., 2022; Carmichael et al., 2022; Paul Friedman et al., 2025).

High-throughput screening (HTS) *in vitro* assays that target a wide range of biological pathways have been developed to efficiently generate data for chemical screening. These assays enable representation of the biology of humans or other species of interest, leading to a better mechanistic understanding of how an adverse outcome is likely to occur. The integration of HTS data for prioritization and into weight-of-evidence-based hazard assessments is gaining traction as HTS assays can help contribute to hazard characterization. For example, the U.S. Environmental Protection Agency (EPA) includes HTS assays in the list of accepted NAMs. Although these activities have demonstrated how NAMs can offer a way to better evaluate mechanistic perturbations, it is often hard to relate these data to endpoints traditionally used for chemical assessments. Adding to the challenge, HTS assays are often conducted in 96- to 384-well plates, resulting in the rapid generation of overwhelming amounts of data housed within disparate data sources with different data structures, reporting formats, and data types. This is a particular challenge since the evaluation and application of NAMs requires the combination of multiple types of information.

Therefore, the consistent use of data from these assays to support toxicological and regulatory needs requires infrastructure that incorporates transparent interpretation and standardized documentation. A key aspect to data curation efforts is ensuring that data management practices support accessibility and interoperability, including standardizing the languages used to annotate information, such as the incorporation of controlled vocabularies and structured ontologies. These approaches allow for information to be accessible and interpretable in a more uniform user-friendly and machine-readable format to regulators, researchers, and method developers. Furthermore, a comprehensive quality assessment of data is essential to ensure their robustness and reliability for use in regulatory decision-making. Various publicly available resources have been developed to address this need, which provide curated, high-quality, toxicologically relevant information. These include the National Institute of Health (NIH) Integrated Chemical Environment (NICE), which was created by the National Toxicology Program Interagency Center for the Evaluation of Alternative Toxicological Methods (NICEATM) to support the application, evaluation, and development of NAMs (Bell et al., 2017; Daniel et al., 2022). NICE includes data from the EPA’s Toxicity Forecaster (ToxCast) program and the U.S. federal Toxicity Testing in the 21st Century (Tox21) partnership (Dix et al., 2007) as derived from the invitrodb database (Feshuk et al., 2023). Invitrodb not only provides concentration–response data but also includes an infrastructure housing a plethora of metadata such as technological annotations and Organization for Economic Co-operation and Development (OECD) Guidance Document (GD) 211 structured information to describe detailed assay technology information.

To address common challenges associated with interpreting large HTS datasets, such as inconsistent terminologies and reporting, we created an enhanced curation approach to provide the most reliable and high confidence data. We then annotated this information using structured ontologies to molecular and biological processes and organized these into toxicological endpoints of regulatory interest to support the interpretation of our curated HTS (cHTS) data. Finally, we leveraged OECD Harmonised Templates (OHTs) to serve as a standardized data format for reporting potential chemical effects on human health and the environment. The OHT 201 format was specifically created to report intermediate effects or non-apical endpoints to help foster the integration of NAM information into chemical hazard and risk assessments (Carnesecchi et al., 2023). By reporting robust and well-defined results in a more useable output for regulatory submission, the research presented herein can contribute to more confident use of NAMs data for decision making. The implementation of standardized ontologies will strengthen researchers’ ability to integrate, explore, and analyze large volumes of heterogeneous HTS data, thereby accelerating the assessment of chemical safety and underlying biological mechanisms. Furthermore, this approach can facilitate the broader, international adoption of NAMs in hazard and risk assessment, as the data can be more easily converted programmatically into standardized reporting formats.

## 2 Methods

### 2.1 Data source

ToxCast and Tox21 HTS data and metadata were downloaded from the EPA invitrodb version 4.2 (https://clowder.edap-cluster.com/spaces/66858831e4b0a7c65d17841d, downloaded September 2024). Data from invitrodb include technological annotations (e.g., *assay_design_type, assay_format*, *detection_technology_type*, and *technological_target_type*), custom target information (e.g., *intended_target_type* and *intended_target_family*), and concentration–response modeling data (e.g., winning model, model parameters, benchmark dose (BMD), concentration that causes a half-maximal response (AC50), top of curve, activity concentration at cutoff (ACC), and response units) resulting from ToxCast Pipeline (tcpl) analysis (Filer et al., 2017).

This HTS data source covers a diversity of targets (i.e., nuclear receptors and enzymes), cell systems (i.e., immortalized and primary cell cultures and cell-free systems), and technologies (i.e., high-content imaging, gene expression quantification, cytotoxicity assessment, and reporter gene assays) generated using high-throughput screening test systems. Data were harmonized to facilitate tcpl analysis, which further puts outputs in a standardized format that enables the integration of data from a variety of vendors and technologies. Details on each specific assay endpoint can be reviewed through invitrodb assay annotations (e.g., *cell_short_name* and *assay_throughput* fields) and OECD GD 211 documentation (OECD, 2014; e.g., *experimental system*, *metabolic competence*, and *response* fields). This information can be crucial to understanding not only the output measured but also quality of the test system. Test system details, including replication and types of controls, are examples of the information that can be retrieved and were reviewed to comprehend biological relevance. Although the current efforts do not focus on reviewing this information, some of these data are carried forward for our annotation efforts, and we encourage HTS data users to reference these in-depth resources for additional context into the test systems.

### 2.2 HTS curation workflow

**FIGURE 1:**
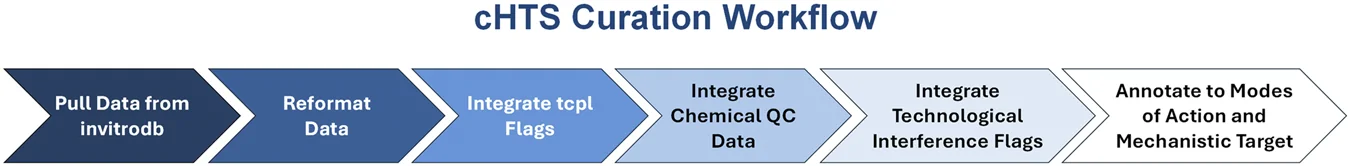
cHTS curation workflow developed for ToxCast/Tox21 assay data. This process starts by retrieving data and metadata from EPA’s invitrodb database. Curators then review information related to assay platforms (invitrodb), concentration–response modeling (invitrodb), chemical quality (NCATS TriPod), and predicted assay technology interference (InterPred) to ensure high confidence data. This includes applying existing curve fitting flags that were determined by the tcpl data analysis pipeline. We then built upon these by using expert knowledge on assay platforms, biological processes, and toxicological pathways to provide additional curation.

To ensure that only the highest confidence ToxCast/Tox21 HTS data are used in hazard assessment and regulatory-ready applications, we have developed a curation workflow that integrates metadata for assay interference, curve-fitting robustness, and chemical QC. This cHTS workflow was developed using the R programming language (R Core Team, 2023 version 4.4.1). Figure 1 depicts the sequential steps of the workflow, which has been previously summarized in Daniel et al. (2022), including (1) assay platform-based technology review, (2) review of concentration–response curve fit parameters, (3) incorporation of chemical quality control (QC) information, and (4) assessment of potential technological interference via InterPred (Borrel et al., 2020). InterPred is a quantitative structure–activity relationship modeling workflow developed to predict luciferase inhibition and autofluorescence under three types of fluorescence. While this workflow was designed using invitrodb version 4.2, it is intended for use with future releases and is a versioned workflow that allows for updates.

#### 2.2.1 Review of assay platform and directionality of response to ensure biological relevance for toxicological interpretation

Because the primary interest in curating HTS assay data is to identify assay endpoints from which mechanistic information can be mapped to biological and toxicological contexts relevant to regulatory endpoints, assay platforms were reviewed for relevance. Instances by which these interpretations were not warranted were excluded from the cHTS dataset. Assays not considered in this analysis include

- zebrafish assays that are currently out of scope for mapping to regulatory endpoints;
- assay endpoints representing readouts that are not meant to be interpreted alone but in tandem with other readouts (e.g., only the Tox21 ratio of channel readouts are included);
- assay endpoints intended to be interpreted in one direction were only summarized in cHTS data if the appropriate direction was the result (e.g., transcriptional activation assay endpoints analyzed in the down direction were excluded such as Attagene transactivation and NovaScreen enzyme inhibition assay endpoints analyzed in the up direction);
- all background readout assays such as fluorescent artifact detection (e.g., TOX21_AutoFluor assay endpoints); these are identified in invitrodb by having the term “background measurement” in the *intended_target_family* field or the term “background control” in the *assay_function_type* field;
- extra runs of assay endpoints that were conducted on chemical subsets or higher concentrations during assay optimization were removed despite being available in the invitrodb assay inventory (e.g., a selection of a few Stemina assay endpoints where the maximum testing concentration was >300 μM).

#### 2.2.2 Review of curve fit and tcpl flags to identify high confidence concentration–response data

Concentration–response curve fitting summary metrics and metadata were reviewed, requiring the retrieval of assay- and endpoint-related fields from invitrodb multi-concentration processing levels 4–6 for all assay endpoints. To curate hit calls, we first defined rules to programmatically assign “FLAG-OMIT” where flagging or assay-specific information may lower the confidence of a tcpl positive hit call. For example, concentration–response curves yielding negative hit calls (indicating that the direction of the curve did not match the intended signal direction) or results where no model could be fit the concentration–response data. Furthermore, if the tcpl level 6 data contained four or more flags for any concentration–response curve, a “FLAG-OMIT” outcome overwrote any active calls from tcpl. This decision was based on interpretation guidance provided for invitrodb data in the official database release notes (US EPA, 2024). In addition to utilizing the tcpl flags which are included in Supplementary Material 1, other instances related to specific assay technologies or winning curve fit models were also defined based on subject matter expertise for assigning “FLAG-OMIT” outcomes as follows:

- NovaScreen cell-free biochemical assays that were active but have less than 50% efficacy;
- any active calls from Tox21 assays where only the lowest concentration tested exceeded the assay activity cutoff threshold and the best-fit curve was a gain–loss model;
- any down-direction (i.e., inhibition, antagonism, and loss-of-signal) active call where the best-fit curve was a gain–loss model;
- any active call where the best-fit curve was a gain–loss model and AC50 was extrapolated below the tested concentration range.

#### 2.2.3 Review for chemical quality to identify chemical purity and concentration

To ascertain chemical quality control (QC), analytical chemistry information for the Tox21 chemical inventory was gathered from the National Center for Advancing Translational Sciences (NCATS) Tripod Tox21 Data Browser (https://tripod.nih.gov/tox/; accessed in March 2021). This information contains assigned chemical grades based on analytical chemistry measurements taken at two different time-points: (1) when the chemical was first taken out of the freezer for an experiment and (2) after storage at room temperature for 4 months. We interpreted these grades, taking into consideration the different time-points, to decide whether samples for each chemical were acceptable or if all data arising from those chemical samples should be flagged “QC-OMIT” (QC score interpretation is summarized in Supplementary Material 1). If a chemical sample did not have QC information in the Tripod database, we extrapolated based on chemical identity according to the QC status of other samples for that chemical based on DTXSID.

#### 2.2.4 Review predicted technological interference

Chemicals were examined to identify instances where chemical structures may interfere with detection technologies, focusing on fluorescence and luciferase technologies. To identify assays that use luciferase and fluorescence technologies, we reviewed assay method descriptions within invitrodb v4.2, assay information, protocols on the NCATS Tripod website (accessed May 2025), and relevant references for each assay. If an assay used fluorescence, additional review was conducted to determine the wavelength or color of fluorescence. To identify potential interferent chemicals, we used data from Tox21 assays specifically designed to test for luciferase inhibition and autofluorescence interference for red, blue, and green wavelengths (Borrel et al., 2020). For the approximately 500 ToxCast chemicals that were not tested in the Tox21 interference assays, we used the InterPred tool to predict luciferase and fluorescence interference (Borrel et al., 2020). InterPred is a quantitative structure–activity relationship modeling workflow developed to predict luciferase inhibition and autofluorescence under blue, green, and red fluorescence, and these predictions were only used where experimental data were not available. Any detected bioactivity resulting in concentration–response curves for a chemical in an assay where it may potentially interfere were flagged.

### 2.3 Annotations and knowledge organization structure

**FIGURE 2:**
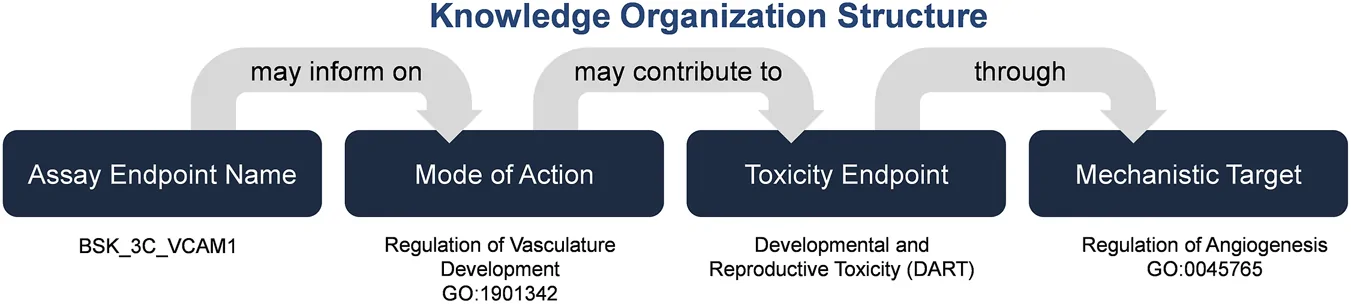
Annotation knowledge organization structure schematic. Assay endpoint names (element 1) gathered from EPA’s invitrodb (v4.2) were annotated with OBO Foundry ontology terms to Mode of Action (element 2) and linked to toxicity endpoints of regulatory concern (element 3), which were in turn mapped to Mechanistic Target (element 4). Most Mechanistic Targets were closely aligned with invitrodb intended targets, but a higher level of biological organization was obtained using OBO Foundry’s ontological terminology for processes. This illustration provides an example of an assay endpoint, BSK_3C_VCAM1, retrieved directly from invitrodb v4.2, annotated to Mode of Action Regulation of Vasculature Development, which may contribute to the Developmental and Reproductive Toxicity endpoint through the Mechanistic Target Regulation of Angiogenesis.

The annotation process started by reviewing metadata fields provided by invitrodb related to the intended molecular target, such as *intended_target_family*, *intended_target_family_sub*, and *biological_process_target*. We then annotated each assay endpoint to a “Mode of Action” term and “Mechanistic Target” term using structured vocabularies. These included existing annotations utilizing the National Cancer Institute Metathesaurus (NCIm, https://ncim.nci.nih.gov/ncimbrowser/) gathered from NICE v4.0.2 and BioAssay Ontology (BAO) annotations within invitrodb. Ontological terms available through the Open Biological and Biomedical (OBO) Foundry (Jackson et al., 2021), which houses a suite of interoperable ontologies that follow best practice principles to ensure open data availability, common language formats, and proper documentation (http://obofoundry.org), were used for our annotation schema. The Mechanistic Target term is reflective of the biological process that the assay target can inform using biochemical and biological terms and ontologies, such as histone modification. The Mode of Action term is related to broader pathways that are relevant to toxicological interpretations which can aid in understanding perturbations that may contribute to adversity, such as the epigenetic regulation of gene expression. To facilitate linkages across these multiple concepts, we have established a knowledge organization structure (Figure 2). In this structure, the Mode of Action is mapped to a “Toxicity Endpoint” of regulatory interest (i.e., acute lethality, endocrine, developmental and reproductive toxicity), which may be impacted through the Mechanistic Target.

Our overall goal in using a controlled vocabulary to populate Modes of Action and Mechanistic Targets was to promote interoperability in widely used and established terminology, thus ensuring that data objects align with data guidelines that enable information to be findable, accessible, interoperable, and reusable (FAIR) to manual and computational approaches. The organized annotations incorporating ontologies were primarily obtained from the Gene Ontology knowledgebase within the OBO Foundry (The Gene Ontology Consortium et al., 2023). Whenever possible, terms within the same ontology tree were selected for Mode of Action and Mechanistic Target to further facilitate computational linkages between these concepts and build a hierarchy for the terminology used.

Since orthologous and complementary cHTS assays are frequently considered in sum to build confidence and contextualize results rather than in isolation on a per assay basis, we endeavored to avoid overly specific terminology in order to facilitate the groupings. This approach avoids forcing complex interpretations on users that would require extensive subject matter knowledge while facilitating assay grouping within Mechanistic Target and Mode of Action terms. After each round of annotation was completed, subject matter experts reviewed and confirmed that appropriate terms were applied.

Our annotation also considered the direction of the assay endpoint’s concentration–response curve, since an increased or decreased response can change the biological interpretation. In instances where curve direction was influential for the biological interpretation, a chemical-assay pair was not annotated if the curve’s direction could not be determined or was not intended to be interpreted in a certain direction.

To gather sufficient information on the specific toxicological modes of action, relevant peer-reviewed literature was reviewed, including literature focused on known effects contributing to acute systemic toxicity (Hamm et al., 2017; Prieto et al., 2019) and endpoints related to teratogenic mechanisms and developmental and reproductive toxicity (DART) (van Gelder et al., 2010) as well as vasculature development (Kleinstreuer et al., 2011). These terms were not reviewed for consistency with adverse outcome pathway initiatives (e.g., AOPWiki) but include ontologies that were a part of the creation of a structured AOP knowledgebase to enable future alignment with such approaches (Ives et al., 2017; Mortensen et al., 2025). We included published research that annotated ToxCast/Tox21 assays to toxicological endpoints but utilized classification terms not part of the controlled vocabularies we had selected for the cHTS data. These included key characteristics of carcinogens (Smith et al., 2016; Cuomo et al., 2026 in prep) and key characteristics of cardiotoxicity (Krishna et al., 2021).

### 2.4 Standardized reporting, OHT 201

To support the use of the curated, robust, annotated cHTS data, we explored how to apply a standardized data reporting format that has been adopted by the OECD. The OECD Harmonised Template 201 (OHT 201) was specifically designed with the intent of reporting NAMs data in a consistent format that would support regulatory use. By leveraging annotations from invitrodb in addition to the Mechanistic Target and Mode of Action annotations we developed, we have defined a programmatic approach for populating OHT 201 forms using robust cHTS data.

Overall, the OHT 201 focuses on reporting information to comprehensively describe the method and assay technology details, along with the outcome and interpretation of results. OHTs are designed flexibly with proposed fields where the reporter is free to determine if, how much, and what level of detailed information they want to provide. Therefore, we integrated the assay details from within invitrodb with our cHTS data, including the biological annotations described above. In addition to metadata provided for all assays in invitrodb, we focused our OHT 201 efforts on the assay endpoints for which the EPA has created comprehensive documentation: OECD Guideline Document (GD) 211. The assay endpoints with GD 211 based documentation have more comprehensive information available in a standardized format to support reporting via OHT 201. Thus, to populate the OHT 201 form, we integrated robust, complementary information originating from invitrodb metadata, GD 211 documentation, our knowledge organization structure terminology, and testing outcomes based on our curation workflow-derived cHTS dataset.

Two primary fields within the OHT 201 “Effect Identification” section, *Process*, and *Object*, describe underlying mechanisms behind the assay of interest and include a restricted list of ontology terms, identified as “picklists” by the OECD. The *Process* field is meant to represent the dynamics of the underlying biological system, while the *Object* field describes the subject of the biological effect. To accomplish this, we first populated the *Process* fields with our Mechanistic Target annotations, since these annotations primarily represent the biological process that the assay endpoint informs on. The *Object* field captures the endpoint that the assay is measuring (i.e., receptor, cell surface marker, and neurite outgrowth), which was retrieved from invitrodb fields such as *intended_target_family*.

## 3 Results

### 3.1 cHTS curation

We began this project by retrieving ToxCast/Tox21 HTS concentration–response modeling metrics, tcpl flags, and assay annotations from EPA’s invitrodb v4.2. In total, 1,570 assay endpoints were retrieved. After omitting assays that were out of scope for our cHTS dataset as described in Section 2.2.1 (e.g., background measurements, channel readouts, select zebrafish assays), 1,366 assay endpoints remained. Of these, we were able to map 1,203 assay endpoints to one or more Mode of Action and Mechanistic Target terms. We found that 163 assay endpoints remain unmapped, as an appropriate controlled vocabulary term could not be identified for that particular assay technology. For example, many of these assays measured mRNA gene expression readouts and therefore could not be interpreted solely on the basis of the transcript (Franzosa et al., 2021).

The resulting NICE cHTS dataset contains 9,614 chemicals, representing 161,742 active and 880,396 inactive concentration–response curves across the 1,366 assay endpoints. Note that not all chemicals were tested in all assay endpoints. We flagged 242,516 concentration–response curves (e.g., chemical tested in an assay endpoint) as having an issue related to curve fitting or FLAG-OMIT; 580,402 curves were flagged due to chemical QC or QC-OMIT; 74,012 curves were flagged for potential technological interference (Table 1 and Supplementary Material 1). Of the flags related to curve fitting, directionality of the curve not matching the intended direction of analysis was most frequently identified. For the chemical QC flagging, the majority of flags arose due to extrapolation across the chemical from a sample that failed evaluation at a later time point after having passed Grade A at an early timepoint. “Grade A” refers to chemicals where the molecular weight was confirmed and purity is greater than 90%. Within the most frequently documented flags, the grade at a later timepoint was Grade AC, in which low concentrations were detected. After that, Grade F, indicating an incorrect molecular weight resulting in unreliable biological activity, was the second highest (Supplementary Material 1). Finally, assay endpoints that utilized luciferase technology were flagged the most for potential technical interference flags.

**TABLE 1:** Number of concentration–response curves flagged for curve fitting issues, chemical QC concerns, and potential technological interference between the assay technology and chemical tested. Chemical QCs are a summary based on the grades at the early timepoints. Specific grade combinations can be found in Supplemental File 1.

Flag type	Flag definition	# Of curves
Curve fitting or FLAG-OMIT	AC50 was extrapolated above the highest tested concentration for an assay with an active hit call	52
	ACC was extrapolated above the highest tested concentration for an assay with an active hit call	38
	BMD was extrapolated above the highest tested concentration for an assay with an active hit call	12,282
	Active cell viability assay that was fit with gain-loss winning model	757
	Curve could not be modeled by tcpl	5,213
	Active NovaScreen assay with less than 50% efficacy	2,518
	Best-fit curve for an active hit call was a gain-loss model, and AC50 was extrapolated below the testing concentration range	873
	Best-fit curve was a gain-loss model for an active down-direction curve	4,178
	Direction of curve does not match the intended direction of analysis	194,640
	Polynomial-quadratic (poly2) model is problematic for borderline cases	14,490
	Active Tox21 assay where a gain–loss model was the best-fit curve, and only the lowest concentration tested exceeded the assay activity cutoff	499
	tcpl processing pipeline assigned four or more flags to the curve	15,427
Chemical QC* or QC-OMIT	Grade A at early timepoint. Another grade at late timepoint	97,979
	Grade AC at early timepoint. The same or another grade at late timepoint	30,856
	Grade B at early timepoint. Another grade at late timepoint	5,344
	Grade BC at early timepoint. Another grade at late timepoint	2,265
	Grade C at early timepoint. Another grade at late timepoint	3,209
	Grade CC at early timepoint. Same or another grade at late timepoint	3,905
	Grade D at early timepoint. Same or another grade at late timepoint	29,769
	Grade F at early timepoint. Same or another grade at late timepoint	70,213
	Grade FC at early timepoint. Same or another grade at late timepoint	14,378
	Grade FNS at early timepoint. Same or another grade at late timepoint	38,583
	Grade I at early timepoint. Another grade at late timepoint	2,522
	Grade M at early timepoint. Grade FNS at late timepoint	158
	Grade U at early timepoint. Another grade at late timepoint	6,1963
	Grade Z at early timepoint. Another grade at late timepoint	5,122
	Sample not tested in chemical QC but another sample for the chemical failed	214,136
Technological interference	Blue fluorescence (experimental)	13,105
	Blue fluorescence (predicted)	1,037
	Green fluorescence (experimental)	6,443
	Green fluorescence (predicted)	615
	Red fluorescence (experimental)	300
	Red fluorescence (predicted)	35
	Luciferase (experimental)	53,812
	Luciferase (predicted)	406

* Grades were taken from the Tox21 Toolbox (MW, molecular weight): A = MW confirmed, purity >90%; B = MW confirmed, purity 75%–90%; C = MW confirmed, purity 50%–75%; Z = MW confirmed, no purity information; AC = caution, low concentration (5%–30% of the expected concentration); BC = caution, low concentration (5%–30% of the expected concentration); CC = caution, low concentration (5%–30% of the expected concentration); D = caution, purity <50%; FC = caution, very low concentration (<5% of the expected concentration) (biological activity unreliable); F = caution, incorrect MW (biological activity unreliable); FNS = caution, no sample detected (biological activity unreliable); I = isomers (two or more isomers detected); M = defined mixture (two or more compounds); U= unknown/inconclusive.

### 3.2 cHTS annotations linked to toxicity endpoints of regulatory interest

Approximately 1,090 assay endpoints were identified that require consideration of response direction to correctly conduct toxicological interpretation. OBO Foundry annotations, including notations on direction of curve, Mode of Action, Mechanistic Target, and Toxicity Endpoints, can be found in Supplementary Material 2. The current cHTS inventory includes assay endpoints mapped to 152 Mechanistic Target terms, 69 Mode of Action terms, and five Toxicity Endpoints. Instances where additional details were needed to resolve annotations, peer-reviewed literature, or resources such as the National Library of Medicine’s Gene resource were used (https://www.ncbi.nlm.nih.gov/gene/, last accessed on March 2024). For example, if an assay’s metadata denoted the intended target family as “immunoglobulin CAM,” peer-reviewed literature was queried and reviewed to relate this to a process or pathway such as *Regulation of Angiogenesis* or *Regulation of Vascular Development* (Polverini, 1996; Francavilla et al., 2009).

**FIGURE 3:**
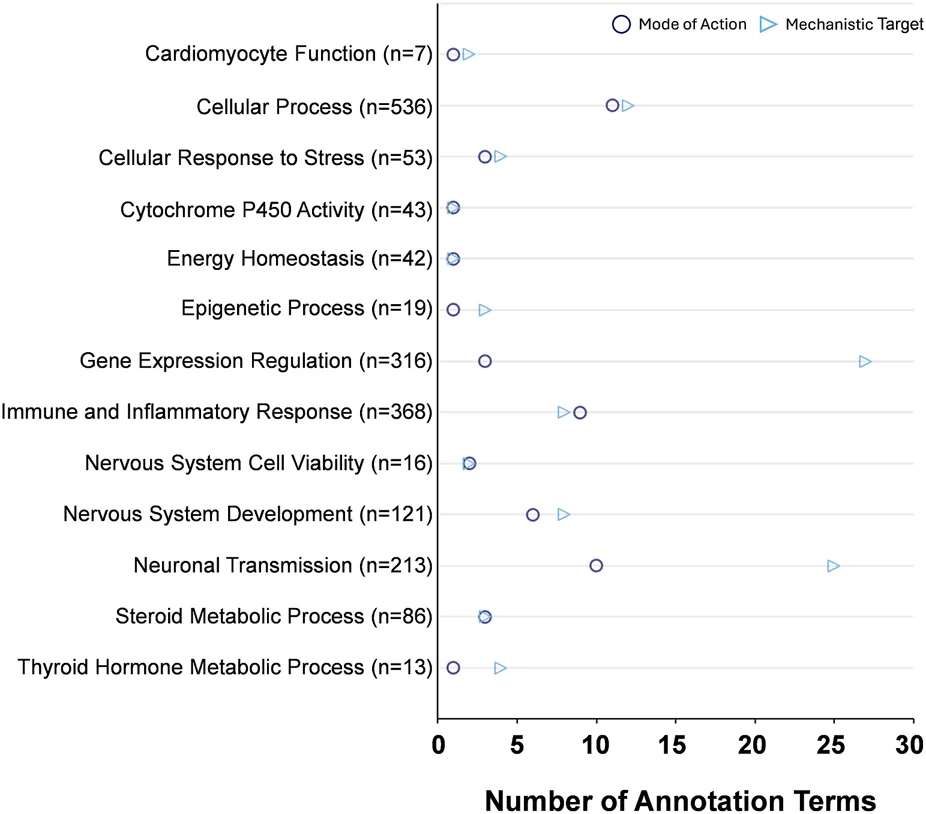
Number of unique OBO Foundry annotation terms for Mode of Action (dark blue circle) and Mechanistic Target (light blue triangle) per general annotation groupings used in the main Assay Selection window within NICE to aid in user selection. The number of assay endpoints within these groupings are in parenthesis. Overlap of circles and triangles indicate that the same number of terms, and often the same terms, were selected for Mode of Action and Mechanistic Target.

Terms selected for Mechanistic Target were slightly more detailed to better recapitulate biological processes, as exemplified by the larger inventory of unique terms integrated when compared to the Mode of Action terms (Figure 3). The largest grouping of assay endpoints (n = 536) was in the Mode of Action term *Cellular Process*, which encompasses assay endpoints related to the Mechanistic Targets *Cell Development*, *Cell Differentiation*, *Regulation of Cell Cycle*, and *Cell Viability Processes*. Conversely, assay endpoints relating to cardiomyocyte function (n = 7) and thyroid hormone metabolic processes (n = 13) contained the least number of associated assay endpoints.

There were instances where the same term was used to represent both a Mode of Action and a Mechanistic Target (Figure 3). For example, assay endpoints relating to *Cytochrome P450* were not further annotated to specific types as this could hinder interpretation by a naive user and were not more generalized for the Mode of Action since these represent a well-known superfamily of metabolizing enzymes. The most diverse groupings, noted by the number of terms and differences between Mode of Action and Mechanistic Targets terms, were related to *Gene Expression Regulation* and *Neuronal Transmission* (Figure 3).

**FIGURE 4:**
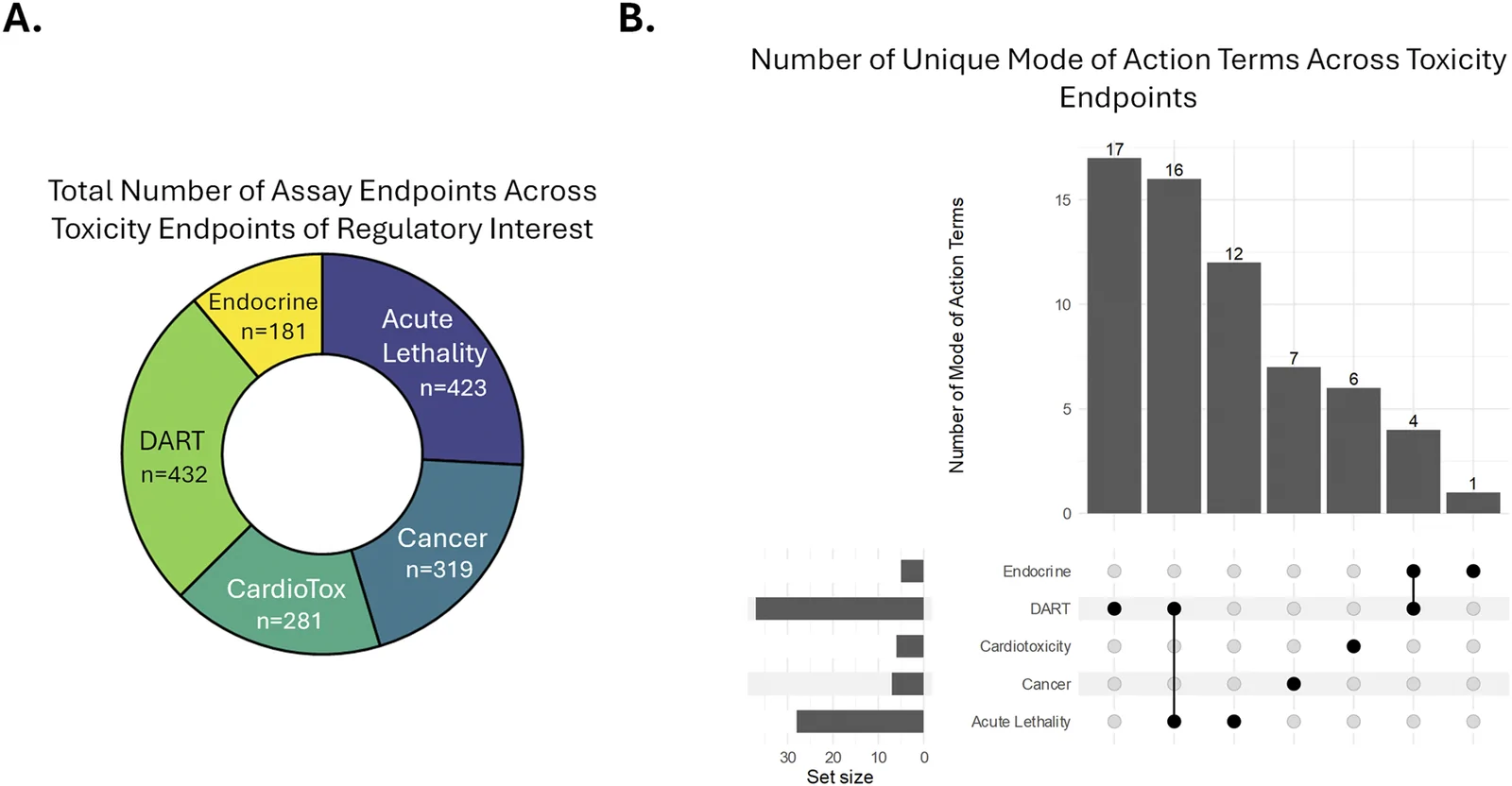
Chart depicting **(A)** the number of unique cHTS assay endpoints and **(B)** number of unique Mode of Action terms within each of the Toxicity Endpoints, chosen based on endpoints of regulatory interest. Overlaps between multiple Toxicity Endpoints are represented by the straight lines connecting the dots underneath the bar plot. These indicate that the same Mode of Action term was mapped to multiple Toxicity Endpoints. Groupings to each Toxicity Endpoint were based on information from peer-reviewed reports of efforts to map ToxCast/Tox21 HTS assays to toxicological endpoints. These include two groupings—Cancer and Cardiotoxicity—that utilized classification terms that were not part of the controlled vocabularies selected for our cHTS annotation scheme.

All cHTS assay endpoints within our dataset (n = 1,203) are mapped to five Toxicity Endpoints of regulatory interest: acute lethality, cancer, cardiotoxicity, developmental and reproductive toxicity (DART), and endocrine (Figure 4A). The Endocrine Toxicity Endpoint is subdivided into androgen, estrogen, thyroid hormone, and other steroid hormones based on hormonal pathways and systems. There is an even representation of cHTS assay endpoints among the different Toxicity Endpoints, particularly between acute lethality and DART, which have a high degree of overlap (Figure 4B). The overlapping terms between these included 16 Mode of Action annotation terms related to *p53 Signaling Pathway*, *Cellular Response to Oxidative Stress*, *Neuron Differentiation*, and *Regulation of Nervous System Process* (Supplementary Material 2, Sheet 2). There is also overlap between endocrine and DART (Figure 4B): all of the assay endpoints mapped to the estrogen (n = 72) and androgen (n = 55) Toxicity Endpoints are also mapped to the DART Toxicity Endpoint, as did most of the other steroid hormone assay endpoints (n = 46 and 78% of total), particularly those annotated to terms such as *Regulation of Hormone Levels*. These overlaps are not surprising due to the involvement of endocrine system pathways such as *Estrogen Receptor Activity* and *Aromatase Activity* in developmental and reproductive processes.

### 3.3 Data availability through NICE

To increase accessibility and facilitate data use, we have made our entire cHTS dataset publicly available through NICE (https://ice.ntp.niehs.nih.gov/). Information for the 9,614 chemicals and 1,203 annotated assay endpoints can be accessed two different ways: zip folder bulk download and representational state transfer (REST) application programming interface (API). The downloadable zip folder is accessible via the Data Sets Page (https://ice.ntp.niehs.nih.gov/DATASETDESCRIPTION). This download features all the information related to chemical QC flags from our workflow (ChemQC Excel File), curve fitting flags (cHTS_FlagOmits Excel File), and technological interference (cHTS_InterferenceFlags Folder and text file). The full suite of concentration–response modeling results can be explored in the text file within the cHTS_DataDownload folder. Alternatively, the REST API can be used, which enables the retrieval of data outside the NICE interface (https://ice.ntp.niehs.nih.gov/api/v1/search). Specific details on accessing the REST API can be found on the NICE User Guide page (https://ice.ntp.niehs.nih.gov/USERGUIDES), but queries can be generally submitted to the Curve Surfer tool that allows the retrieval of concentration–response data from the cHTS dataset. We have included the annotations as Supplementary Material 2.

### 3.4 Adoption of annotations to OHT 201

To further facilitate the application of our cHTS in hazard and risk assessment, we defined an approach to populate OHT 201 templates to provide a standardized way of reporting non-apical intermediate effects (i.e., mechanistic information) that supports regulatory review and submission. We refined our list of cHTS assay endpoints amenable to OHT 201 generation to 986 based on available OECD GD 211-based documents curated by the EPA, ensuring that the robust documentation of assays was available to support reporting readiness. Because the OHT 201 form includes fields that describe the mechanisms measured by assays of interest with a restricted list of ontology terms, identified as “picklists” by the OECD, our starting point for this research was the Mechanistic Target OBO Foundry annotations (Table 2). We were able to utilize 11 of the existing picklist terms from the OHT 201 *Process* field to annotate approximately 43% of cHTS assay endpoints, primarily annotating assays to the Mechanistic Targets *Gene Expression, Receptor Activity,* and *Signaling*. The OHT 201 *Object* field, which describes the subject of the biological effect, proved challenging to map to existing terms due the wide range of biological coverage the cHTS assays encompass. We were able to align only 83 cHTS assay endpoints to existing terms within the OECD picklist for *Object*. However, it should be noted that neither the *Process* nor the *Object* terms are required by OECD, and their recommendations advise that only one of these fields be populated. There were some assay endpoints for which we did not identify terms for the *Object* field due to the technology of the assay. Because the *Object* field is intended to represent the subject of the biological effect observed, it can be difficult to relate it to annotations based on the assay technology. For example, assays that measured cell counts were annotated to *Cell Proliferation* in the *Process* field, but we were unable to annotate these to the *Object* field because of the lack of a specific biomarker for this endpoint (Supplementary Material 2, Sheet 4). Our experience in mapping our annotated cHTS assay data to the OHT 201 templates suggests that there is a need to broaden the OHT 201 picklist terms to include more biological coverage or structure these fields such that the submitter has the option to populate free text fields within a preferred or specified controlled vocabulary.

**TABLE 2:** Examples of the translation between the Mechanistic Target annotation terms and OHT 201 *Process* and *Object* fields. *Process* and *Object* fields are intended to describe the mechanism that can be measured using the assay. At the time of publication, some of the selected terms were not part of the template picklist. Blank spaces indicate that a term was not selected.

Mechanistic target term	OHT 201 *Process* term*	OHT 201 *Object* term*
Energy homeostasis	Energy homeostasis	Cell morphology
Extracellular matrix organization	Extracellular matrix organization	Matrix metalloproteinase
Neural precursor cell proliferation	Cell proliferation	​
p53 signaling pathway	Signaling	p53 protein
Regulation of angiogenesis	Regulation of angiogenesis	Vascular cell adhesion protein 1
Thyroxine deiodinase activity	Enzyme activity	Type III thyroxine deiodinase

* These terms may or may not be included in the current inventory of the pick-list. Our research supports diversifying the inventory of available terms; thus, these standardized fields may be subject to future refinement.

Of the 70 fields in the OHT 201 form, we have mapped data, annotations, and metadata to 58 fields to support robust documentation of cHTS results. The GD 211-based documents available through invitrodb were imperative to organizing and populating these fields, such as information on the test system and method details. For example, *Assay Summary*, *Scientific Principles*, *Metabolic Competence*, and *Exposure Regime* within GD 211 sections were used to populate the OHT 201 fields *Principle of Method, Metabolic Competence of the Test System*, and *Experimental Conditions*. Details on the response (i.e., AC50, BMD, and unit) were also included from tcpl and our cHTS workflow. Concentration–response curves and corresponding curve fitting parameters (e.g., top of curve and ACC) will be provided. Since these forms are intended for robust activities such as regulatory submission, only active responses with no flags were used to create OHT 201. Altogether, we have standardized the reporting of curated and annotated information using a globally recognized and reliable template to make these data available in a retrievable format ready for use and submission.

## 4 Discussion

The rapid development of HTS assays is increasing the availability of diverse mechanistic data for thousands of chemicals. However, there is a need for ways to ensure that these data are robust and easily interpretable for application in regulatory applications. Without the ability to put these data into more biological and regulatory contexts, the pace of NAM adoption will be hindered. To aid with the interpretation of data, it is also imperative that information such as how an assay was conducted and how data were analyzed is available in a harmonized or standardized format. This further enables information to be reproducible and machine readable, facilitating the use of automated ways to gather information and develop computational tools.

A key challenge to the use of HTS programs, such as Tox21/ToxCast, is the sheer volume of not only endpoints but types of assays and assay sources (e.g., vendors) represented. Both Tox21 and ToxCast have great resources for documenting assay systems as well as resultant data. The Tox21 Tripod and ToxCast invitrodb serve as valuable resources for end users to access key information pertaining to assay conduct, quality, and control data. Ultimately, the review of such information by end users is important when resultant data are being integrated into a chemical assessment, as outlined by the Interagency Coordinating Committee on the Validation of Alternative Methods (ICCVAM) Validation Workgroup recommendations that emphasize defining fit-for-purpose assay documentation when applying non-guideline assays (ICCVAM, 2024). Such information may include cell-line identity and authentication, contamination testing, passage number and passage limits, media conditions, seeding density, inclusion of cell viability testing, vehicle and positive control performance, variability analysis (within plate and between laboratories), replication, target expression or phenotype information, and metabolic competence. While this study offers structure to the resultant data in support of facilitating data retrieval and robust outcome reporting, it is critical that this “background” information pertaining to the quality of the data being used by end-users also be considered.

Generating an abundance of screening data using high-throughput screening assays presents novel insights but also novel challenges. While analysis pipelines have been developed to handle this massive volume of data (i.e., tcpl), applications within a regulatory context require additional scrutiny. Thus, we have created a curation pipeline to help identify the most robust data and offer transparent flagging to demark data that require additional review. Our curation pipeline is complementary to EPA’s tcpl as it retrieves outputs from the analysis pipeline and applies additional subject-matter-expert defined rules, integrates concentration–response curve fit flags, considers chemical QC, and notes technological interference. The resulting cHTS dataset is one that is ready for regulatory consideration.

The mechanistic and pathway insight gathered from HTS data can be harnessed to understand specific study types (i.e., acute lethality) or translated to apical endpoints traditionally used for chemical assessments. A multitude of different databases provide and support access, storage, and retrieval of HTS data. However, challenges exist when trying to translate information between these databases due to disparate data structures and reporting initiatives (Watford et al., 2019). To aid the use and interpretation of publicly available ToxCast/Tox21 HTS data in a toxicologically relevant context, we have created a curation and annotation scheme. We demonstrated with our curation workflow that robust mechanistic cHTS data can be appropriately mapped to Toxicity Endpoints in a regulatory context of use, and by using controlled vocabularies we are able to make these interpretations more transparent, interoperable, and standardized. This not only supports data transparency, interoperability, and interpretation but also contributes to reporting and application for regulatory contexts of use.

The appropriate annotation of data is vital to ensuring that NAM data can be integrated into safety assessments. To ensure that our annotation process would support data interoperability and align with FAIR principles, we adhered to standardized ontology terms and harmonized nomenclature for the cHTS annotations. The OBO Foundry ontology has been used in multiple curation studies and computational approaches similar to ours, including semantic data mapping and generating knowledge graphs (Alkhamisi and Saleh, 2020). These ontology terms have also been proposed for integration within adverse outcome pathways, which facilitate application of NAMs by providing a conceptual framework for creating integrated approaches to testing and assessment that allow data from multiple sources to be combined to inform a toxicity endpoint (Ankley and Edwards, 2018; Karmaus et al., 2025). Ontology terms are increasingly the focal point for integration in harmonized data reporting templates (Ives et al., 2017; Carnesecchi et al., 2023). Previous annotation schemes (e.g., National Cancer Institute Metathesaurus (NCIm) and BAO) have been investigated to relate cHTS data into biomedical contexts (Supplementary Material 2, Sheet 3). While these provided useful clinical and cancer focused annotations, expansion to incorporate the OBO Foundry was undertaken to relate information to a broader biological and toxicological space and enable more refined annotation for some Toxicity Endpoints that were not within the scope of NCIm, such as Neurotransmission within DART (Supplementary Material 2, Sheet 3). OBO Foundry and NCIm annotations are provided as part of the Supplementary Materials to enable broad use of our cHTS data and acknowledge that the cHTS dataset in NICE utilized this annotation until version 4.0.2, when the OBO Foundry annotations summarized herein were adopted. Thus, it should be noted that the latest version of NCIm annotations were conducted using a previous version of invitrodb (and thus cHTS dataset). Altogether, this annotation scheme combined with the technical assay information that can be gathered through the invitrodb database broadens the applicability of cHTS data for assessments and increases interoperability with other resources (e.g., computational approaches).

Ensuring that curated data can be accessible and retrievable in multiple formats is crucial for the application of NAMs*.* This cHTS dataset has been made available through NICE (https://ice.ntp.niehs.nih.gov/), a free online resource created to support the development and evaluation of NAMs with regulatory applications in mind. We structured our cHTS into a knowledge organization structure focused on linkage to Toxicity Endpoints that support visualizations and groupings by Mechanistic Target, Mode of Action, and ultimately Toxicity Endpoint. Structuring and annotating data in this way allows complex mechanistic data to be more approachable, based on the toxicological context of use defined by the end-user. A recent demonstration of a cHTS use case within NICE is described by Unnikrishnan et al. (2026) in prep.

**FIGURE 5:**
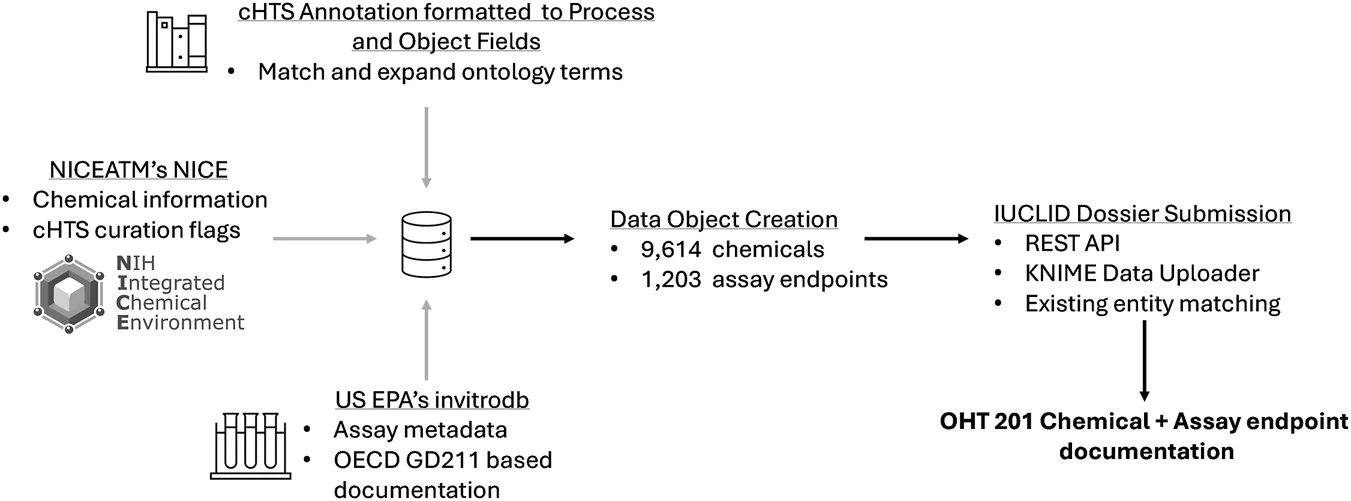
We are developing an automated and semiautomated workflow to populate the OECD Harmonised Template 201 (OHT 201) with our cHTS data. HTS assay data obtained from EPA’s invitrodb 4.2 were curated and annotated as described in “Methods”. We then mapped these data to the OHT 201 Process and Object fields. The International Uniform Chemical Information Database (IUCLID) Dossier Submission step represents a future direction, detailed in “Discussion”.

To support the reporting of the cHTS data in a standardized format, we have aligned annotations and metadata to OHT 201 to facilitate integration of non-guideline NAM data in weight-of-evidence and regulatory contexts. Defining how to populate an OHT 201 for cHTS data is the first step in bringing this massive compendium to the next level (e.g., standardized reporting of curated, annotated, regulatory submission-ready HTS data). We have utilized information from invitrodb, our own annotations, and the additional literature reviews on assay details to comprehensively ensure that detailed data are mapped to the OHT 201 fields. A challenge we encountered was the limited number of terms in the current OHT 201 picklists, which may stem from the unprecedented effort to incorporate thousands of different chemical-assay endpoints. While OHT 201 in its current form captures a variety of mechanistic information (Carnesecchi et al., 2023), this was to our knowledge the first study to populate these templates with thousands of chemicals and assay endpoints, which would be greatly aided by developing a way to automate the process. The OHTs are primarily structured for regulatory use and will aid confidence in the use of NAM data. Furthermore, the detailed information provided in OHTs on the technical aspects and interpretation of assays are also beneficial for toxicology and biomedical applications. At the time of writing, we are in the process of creating a semi-automated workflow to populate OHT 201 fields for upload into the International Uniform Chemical Information Database (IUCLID)—a repository for OHTs (Figure 5). The expansion of the number of terms available on the picklist and the harmonization of those terms with broadly used controlled vocabularies and ontologies such as NCIm and OBO Foundry will reduce the need for users to choose the “other” picklist option and manually enter the annotations into the Remarks field. Requiring users to manually input such information hinders the ability of subsequent users to search for specific annotation details or similar dossiers within IUCLID.

In conclusion, the adoption of NAM data, including the interpretation of HTS data, remains a challenge in regulatory contexts. To overcome this challenge, we created a workflow focusing on a publicly available robust cHTS dataset by structuring and annotating these to regulatory toxicity endpoints of interest. We plan to continue to refine cHTS annotations based on NICE user feedback and new updates to invitrodb and will develop annotations and curation as more data are generated and more targets are defined (e.g., new evidence to suggest linkage between mode of action and adverse events). To further highlight the regulatory readiness of these and to make data more transparent and interoperable, we leveraged these cHTS assays to generate globally recognized templates created to define results into a more useable output for regulatory submission. Future research will focus on expanding the population of OHT 201 by integrating additional cHTS information into the IUCLID database.

## Data Availability

The datasets presented in this study can be found in online repositories. The names of the repository/repositories and accession number(s) can be found in the article/Supplementary Material.

## Supplementary material

SUPPLEMENTAL TABLE S1 Information regarding curation flags applied to the high-throughput screening data set, including the number of curves per flag.

SUPPLEMENTAL TABLE S2 Annotations for the curated high-throughput screening data set, including structured ontology and controlled vocabulary terms.
